# Regulated inositol synthesis is critical for balanced metabolism and development in *Drosophila melanogaster*

**DOI:** 10.1242/bio.058833

**Published:** 2021-10-28

**Authors:** Maria J. Rivera, Altagracia Contreras, LongThy T. Nguyen, Elizabeth D. Eldon, Lisa S. Klig

**Affiliations:** Department of Biological Sciences, California State University Long Beach, Long Beach, CA 90840, USA

**Keywords:** Developmental defect, Obesity, Inositol, Metabolism, Proboscis, Head

## Abstract

*Myo*-inositol is a precursor of the membrane phospholipid, phosphatidylinositol (PI). It is involved in many essential cellular processes including signal transduction, energy metabolism, endoplasmic reticulum stress, and osmoregulation. Inositol is synthesized from glucose-6-phosphate by *myo*-inositol-3-phosphate synthase (MIPSp). The *Drosophila melanogaster Inos* gene encodes MIPSp. Abnormalities in *myo*-inositol metabolism have been implicated in type 2 diabetes, cancer, and neurodegenerative disorders. Obesity and high blood (hemolymph) glucose are two hallmarks of diabetes, which can be induced in *Drosophila melanogaster* third-instar larvae by high-sucrose diets. This study shows that dietary inositol reduces the obese-like and high-hemolymph glucose phenotypes of third-instar larvae fed high-sucrose diets. Furthermore, this study demonstrates *Inos* mRNA regulation by dietary inositol; when more inositol is provided there is less *Inos* mRNA. Third-instar larvae with dysregulated high levels of *Inos* mRNA and MIPSp show dramatic reductions of the obese-like and high-hemolymph glucose phenotypes. These strains, however, also display developmental defects and pupal lethality. The few individuals that eclose die within two days with striking defects: structural alterations of the wings and legs, and heads lacking proboscises. This study is an exciting extension of the use of *Drosophila melanogaster* as a model organism for exploring the junction of development and metabolism.

## Abstract (precis)

**Summary:** Inositol reduces obesity and high blood (hemolymph) glucose, but can cause dramatic developmental defects. This study uses the model organism *Drosophila melanogaster* to explore the junction of development and metabolism.

## INTRODUCTION

*Myo*-Inositol metabolism is involved in many biological processes and human diseases. Diabetes, cancer, and many neurodegenerative disorders are often associated with alterations in inositol metabolism (Koguchi et al., 2016; Bevilacqua and Bizzari, 2018; Chhetri, 2019; Guo et al., 2019; D'Souza et al., 2020; Kiani et al., 2021). Reproductive and fertility defects, as well as high-blood glucose and obesity, can be alleviated by dietary inositol supplementation (Croze and Soulage, 2013; Dinicola et al., 2017; Shimada et al., 2019; Wojciechowska et al., 2019). In contrast, diseases of brain development and dysfunction are associated with elevated levels of inositol and some are treated by pharmacological agents that reduce the inositol levels (Yu et al., 2017; Case et al., 2018; Patkee et al., 2021). *Drosophila melanogaster* (fruit fly) was established as a model to study inositol metabolism with a demonstration of its essential role in spermatogenesis (Jackson et al., 2018). This study examines the regulation of inositol synthesis in animals, and its role in metabolism and development in *D. melanogaster*.

*Myo*-inositol, a six-carbon sugar alcohol, is found in eukaryotic and many prokaryotic cells. It is a precursor of the essential membrane phospholipid phosphatidylinositol (PI), and has roles in signal transduction, endoplasmic reticulum stress (unfolded protein response), energy metabolism, nucleic acid synthesis, and osmoregulation (Henry et al., 2014; Basak et al., 2018; Cui et al., 2020).

The three ways an organism can acquire inositol are: (1) transport from the extracellular environment (Spector and Lorenzo, 1975), (2) recycling of inositol phosphates (Berridge and Irvine, 1984; Janardan et al., 2020), and (3) synthesis from glucose-6-phosphate. The first and rate limiting step of inositol synthesis is catalyzed by *myo*-inositol-3-phosphate synthase (MIPSp, EC 5.5.1.4 alternate name *myo*-inositol-1-phosphate synthase) (Loewus and Kelly, 1962; Eisenberg et al., 1964). The physical properties and enzyme catalytic mechanisms of MIPSp have been found to be similar in yeast, plants, and animals (Chen and Charalampous, 1964; Loewus et al., 1983; Klig et al., 1994; Molina et al., 1999; Hazra et al., 2019). Genomes of many organisms, ranging from microbes to humans, contain annotated orthologs encoding MIPSp (NCBI). It is a cytoplasmic enzyme which in most organisms has been shown to be a homo-tetramer (∼62 kDa per subunit) (Majumder et al., 2003). In the model organism *Saccharomyces cerevisiae*, less inositol is synthesized when inositol is available in the environment (Donahue and Henry, 1981; Henry et al., 2014).

*Inos*, the gene encoding *Drosophila melanogaster myo*-inositol-3-phosphate synthase (MIPS), is located on chromosome 2 at band 43C3. An *Inos* cDNA clone was expressed yielding a 565 amino acid MIPS protein with a molecular weight of 62.3 kDa (Park and Kim, 2004). High levels of *Inos* mRNA have been observed in the early embryo before zygotic transcription, the levels again peak at 6-10 h of embryogenesis, and during all three larval stages (Graveley et al., 2011; Thurmond et al., 2019). High throughput analyses reveal that in the adult it is highly expressed in the head and testes (Chen et al., 2014).

*D. melanogaster* development has been extensively studied. In 1 day, after fertilization, the embryos proceed through 17 stages of development during which the imaginal discs are laid out (later to give rise to adult body structures). Maternal contributions to the egg include *Inos* mRNA which is at a high level in pre-cellularization embryos (Pilot et al., 2006). Head involution, which begins 10-12 h after fertilization (Campos-Ortega and Hartenstein, 1985), is coincident with high embryonic *Inos* mRNA levels. During this process the lobes that form the larval head structures rearrange and carry with them the imaginal disc primordia (Younossi-Hartenstein et al., 1993).

After hatching, during the next 4-5 days, *D. melanogaster* larvae feed constantly and proceed through three larval instar stages. Throughout these stages, *Inos* mRNA is expressed (Graveley et al., 2011). Nutritional checkpoints during larval development are important as insufficient energy storage leads to pupal lethality (Pan et al., 2019). Pulses of the steroid hormone ecdysone initiate a cascade of events that stimulates metamorphosis. Metamorphic onset in *D. melanogaster* and pubertal onset in mammals have been described as involving similar processes in which a steroid hormone initiates the juvenile to adult transition (Delanoue and Romero, 2020). During pupal development *Inos* mRNA expression is lower than during larval stages (Graveley et al., 2011). In *D. melanogaster*, pupal development proceeds through fifteen stages (P1-P15) for 4-5 days, after which the adults eclose (Bainbridge and Bownes, 1981). Pharate adults are flies that have largely undergone metamorphosis but have not emerged from the pupal case. Pupal lethality can be caused by mutations in components of the phosphoinositide pathways (PI synthase, PI4 kinase, and PI4P phosphatase) (Del Bel and Brill, 2018; Janardan et al., 2020). The typical lifespan of a *D. melanogaster* adult is 20-60 days.

In recent years *D. melanogaster* has emerged as an excellent model organism for studying metabolic diseases including diabetes (Musselman et al., 2011, 2019; Tennessen et al., 2014; Ugrankar et al., 2015; Williams et al., 2016; Gillette et al., 2021; Graham and Pick, 2017; Mattila and Hietakangas, 2017; Huang et al., 2019). Assays for obese-like phenotypes and hemolymph glucose have been established (Reis et al., 2010; Musselman et al., 2011; Tennessen et al., 2014; Rovenko et al., 2015; Hazegh and Reis, 2016; Musselman and Kühnlein, 2018). An exciting extension of the use of *D. melanogaster* as a model organism for developmental or metabolic studies is to explore the junction of development and metabolism.

In the current study, *myo-*inositol synthesis and its role in growth and development were explored in the model organism *D. melanogaster*. Dietary or endogenously synthesized inositol was shown to reduce both the obese-like and high-hemolymph glucose phenotypes of third instar larvae fed a high sucrose diet. Moreover, this study seems to be the first demonstration in animals that *Inos* mRNA levels are regulated in response to dietary inositol. *D. melanogaster* strains with upregulated, nearly constitutive, *Inos* mRNA levels had a dramatic reduction of the obese-like and high-hemolymph glucose phenotypes in third instar larvae, and also displayed pharate adult developmental defects with high levels of pupal lethality. The few individuals that did eclose died within 2 days with striking defects – there were structural alterations of the wings and legs, and they lacked proboscises. These studies contribute to the understanding of the role of inositol synthesis in metabolism and development.

## RESULTS

### *Inos* mRNA is regulated in response to inositol

**Fig. 1.**
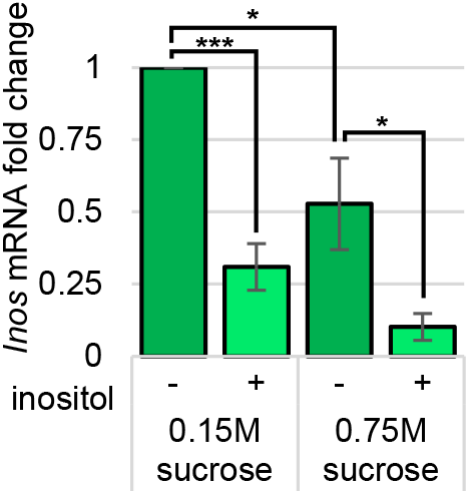
***Inos* mRNA levels are regulated in response to dietary inositol.** qRT-PCR experiments with wild-type Canton-S larvae (*N*≥10 per experimental condition per trial) grown on low- and high- sucrose semi-defined food (50 µM inositol supplementation as indicated). Normalized to RPL32. Mean±s.e. of five independent trials are represented. *P<0.05; ***P<0.0001 as indicated determined by two-tailed *t*-test.

To determine whether the levels of *Inos* mRNA are regulated in response to inositol and/or a high-sucrose diet, qRT-PCR experiments were performed. RNA was extracted from third instar wild-type (Canton-S) *Drosophila melanogaster* larvae grown on a semi-defined low- (0.15 M) or high- (0.75 M) sucrose diet with 0 or 50 µM inositol supplementation. Lower *Inos* mRNA levels were observed in larvae grown on the high-sucrose (0.75 M) diet than the low-sucrose diet (0.15 M). In both cases, the level of *Inos* mRNA was significantly lower when 50 µM inositol was provided (Fig. 1).

### Dietary inositol reduces obesity and hemolymph glucose in larvae

**Fig. 2.**
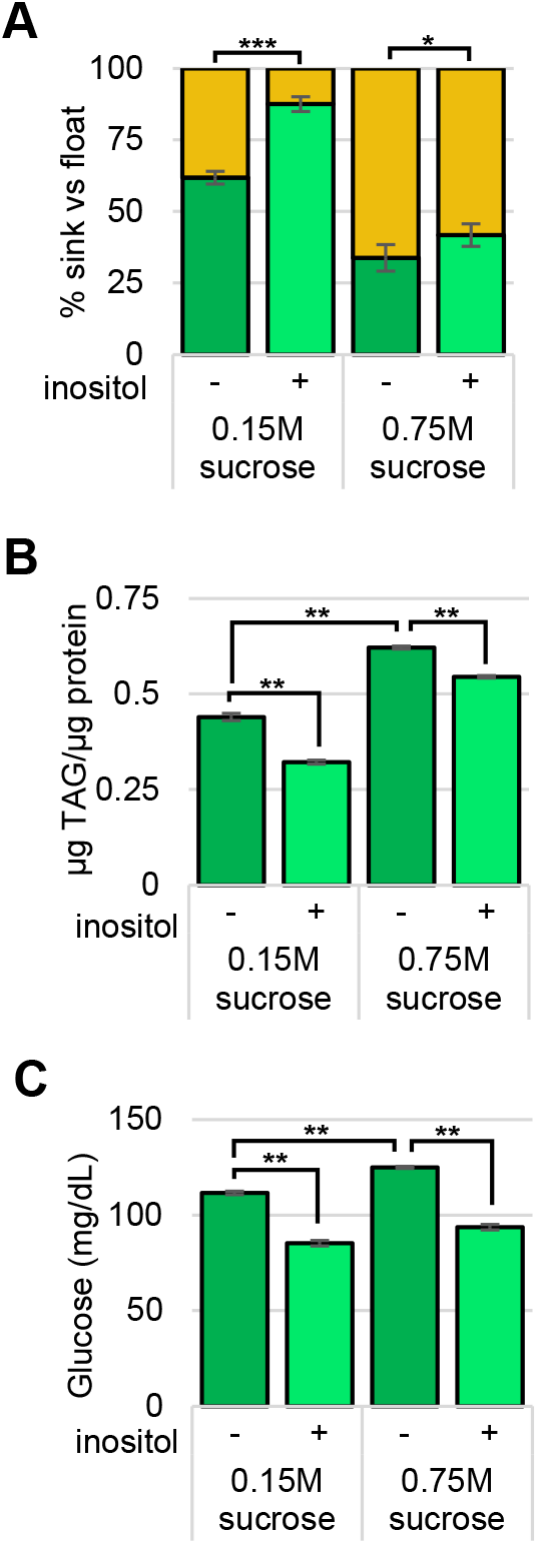
**Dietary inositol decreases larval obesity and reduces hemolymph glucose.** Wild-type Canton-S larvae grown on low- and high- sucrose semi-defined food (50 µM inositol supplementation as indicated). (A) The percentage of larvae that float (gold segment of bars) and sink in a buoyancy assay are indicated. *N*=total number of larvae. (B) Larvae assayed for TAG levels, values indicated are normalized to total protein. *N*=6 per condition per trial. (C) Glucose (mg/dl) in the hemolymph of the larvae. *N*=5 per condition per trial. Mean±s.e. of three independent trials of each experiment are represented. *P<0.05; **P<0.005; ***P<0.0001 as indicated determined by two-tailed *t*-test.

To examine the effect of dietary inositol on obesity and hemolymph glucose, third instar wild-type (Canton-S) *D.* *melanogaster* larvae were again grown on a semi-defined low- (0.15 M) or high- (0.75 M) sucrose food with 0 or 50 µM inositol supplementation. Nearly double the proportion of wild-type (Canton-S) larvae grown on a high-sucrose diet were obese-like (float in buoyancy assay) with higher triglyceride (TAG) and hemolymph glucose levels (Fig. 2), than when grown on a low-sucrose diet. Inositol supplementation reduced the proportion of obese-like larvae and TAG levels for both the low- and high-sucrose conditions (Fig. 2A,B). Moreover, supplementary inositol reduced the concentration of glucose in the hemolymph of larvae on both the low- and high-sucrose diets (Fig. 2C).

Since there was a reduction in obesity and hemolymph glucose when a semi-defined diet was supplemented with 50 µM inositol, the next question addressed was whether these changes would be evident on a standard rich diet. As detailed below (displayed as the control in Fig. 4), float buoyancy, TAG, and hemolymph glucose assays were performed using wild-type (Canton-S) larvae grown on standard rich food supplemented with 0 or 50 µM inositol. Addition of inositol reduced the proportion of obese-like (floating) larvae, the TAG levels, and reduced the hemolymph glucose concentration. It is interesting to note that the proportion of larvae grown on the high-sucrose diet identified as obese-like in the float buoyancy assay is identical to that of larvae grown on standard rich food (Figs 2A and 4A). Moreover, the TAG levels of larvae grown on the high-sucrose diet are the same as the TAG levels of larvae grown on the standard rich food (Figs 2B and 4B).

### Increased levels of *Inos* mRNA and MIPSp decrease larval obesity and hemolymph glucose

**Fig. 3.**
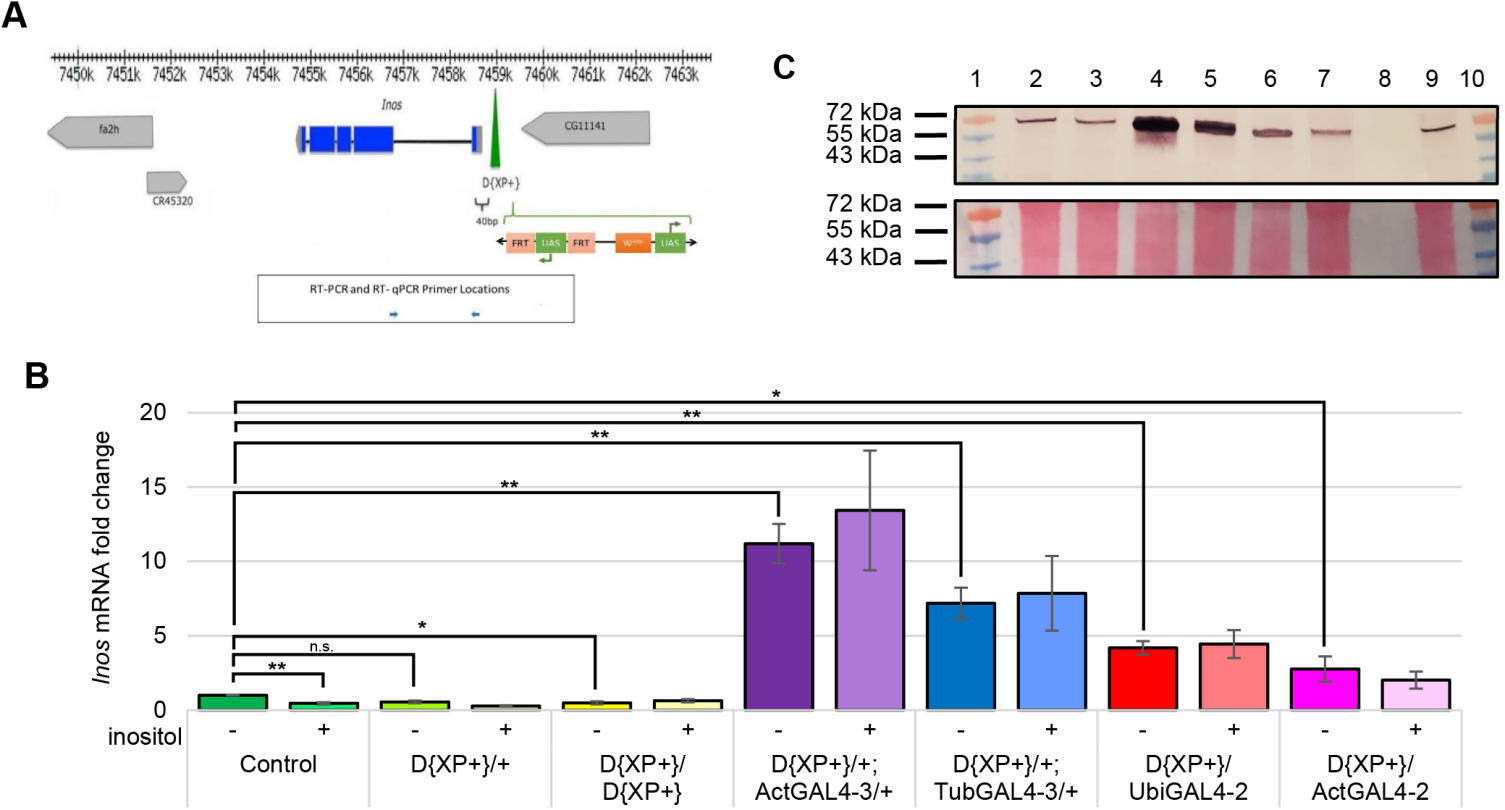
***Inos* mRNA levels vary with promoter-GAL4 constructs and MIPS protein levels vary in concordance with *Inos* mRNA levels.** (A) The *D. melanogaster Inos* gene and the surrounding genomic region of chromosome 2. Location of the P-element D{XP+} is indicated by a green triangle. The primers for exon 1 to exon 2 (qRT-PCR experiments) are blue arrows. The details of the XP+ element are not to scale. Exons are blue blocks, introns are black lines, UTRs are grey blocks. (B) qRT-PCR experiments with larvae (*N*≥10 per experimental condition per trial) grown on standard rich food (inositol supplementation as indicated). Normalized to RPL32. Three independent trials of (control) strain ActGal4-3/+ were indistinguishable from the wild-type control Canton-S results shown. Mean±s.e. of three-six independent trials are represented. *P<0.05; **P<0.005 as indicated determined by two-tailed *t*-test. (C) Western blot of crude lysates extracted from third instar larvae (*N*≥30 per trial) of the indicated genotypes grown on standard rich food (upper panel). Ponceau staining of the blot (lower panel). Lane 1 molecular weight markers, lane 2 Canton-S, lane 3 D{XP+}/D{XP+}, lane 4 D{XP+}/+; ActGAL4-3/+, lane 5 D{XP+}/+; TubGAL4-3/+, lane 6 D{XP+}/UbiGAL4-2, lane 7 D{XP+}/ActGAL4-2, lane 8 blank, lane 9 D{XP+}/+. One representative blot of five independent trials is shown.

This led to another question: would increasing endogenous inositol by altering the expression of the *Inos* gene affect obesity and hemolymph glucose? To address this question, four *D. melanogaster* strains were generated. Each of the strains had one wild-type *Inos* gene and one *Inos* gene with the D{XP+} element inserted immediately upstream (5′). The D{XP+} element contains a UAS_GAL4_ in the correct orientation to promote transcription of the *Inos* gene when the GAL4p is present (Fig. 3A). Each strain also had *GAL4* transcription controlled by of one of three different promoters of highly expressed genes (Actin 5C, Tubulin 84B, and Ubiquitin). To test position effects versus temporal and spatial expression effects, Actin5C-GAL4 constructs in two different locations were used, one on chromosome 3 and the other on chromosome 2 (identified as ActGAL4-3 and ActGAL4-2). The Tubulin84B-GAL4 construct is on chromosome 3 (hereafter referred to as TubGAL4-3) and the Ubiquitin-GAL4 construct is on chromosome 2 (hereafter referred to as UbiGAL4-2). To generate the four strains, all the GAL4 containing strains were marked with GFP via crosses to strains harboring GFP on corresponding balancer chromosomes. These GFP marked GAL4 containing strains were then crossed to the homozygous D{XP+} strain to generate heterozygous progeny with one copy of a GAL4 construct, a wild-type *Inos* gene, and an *Inos* gene with the D{XP+} element upstream. The homozygous D{XP+}/D{XP+} strain has neither GAL4 nor a wild-type *Inos* gene.

To confirm that the levels of *Inos* mRNA were both upregulated and dysregulated, qRT-PCR experiments were performed. As displayed in Fig. 3B *Inos* mRNA levels in the control wild-type (Canton-S) larvae were lower when the standard rich food was supplemented with 50 µM inositol. As expected, *Inos* mRNA levels in larvae with the D{XP+} element and any of three different promoters driving GAL4 production were higher than the wild-type levels. The three highest levels were observed with the two constructs on chromosome 3 (ActGAL4-3 and TubGAL4-3), and one construct on chromosome 2 (UbiGAL4-2). In these strains, *Inos* mRNA levels did not appear to vary significantly with 50 µM inositol supplementation. *Inos* mRNA levels in larvae with the ActGAL4-2 construct on chromosome 2 were lower than the other three constructs, although still higher than the wild-type strain, and even lower with 50 µM inositol supplementation. Interestingly, homozygous D{XP+}/D{XP+} larvae had the lowest levels of *Inos* mRNA, with no significant difference apparent when the diet was supplemented with inositol. Western blot analyses confirmed that a band of the expected size of MIPSp displayed varied intensities that appear to correspond to the observed *Inos* mRNA levels for each of the strains (Fig. 3C).

**Fig. 4.**
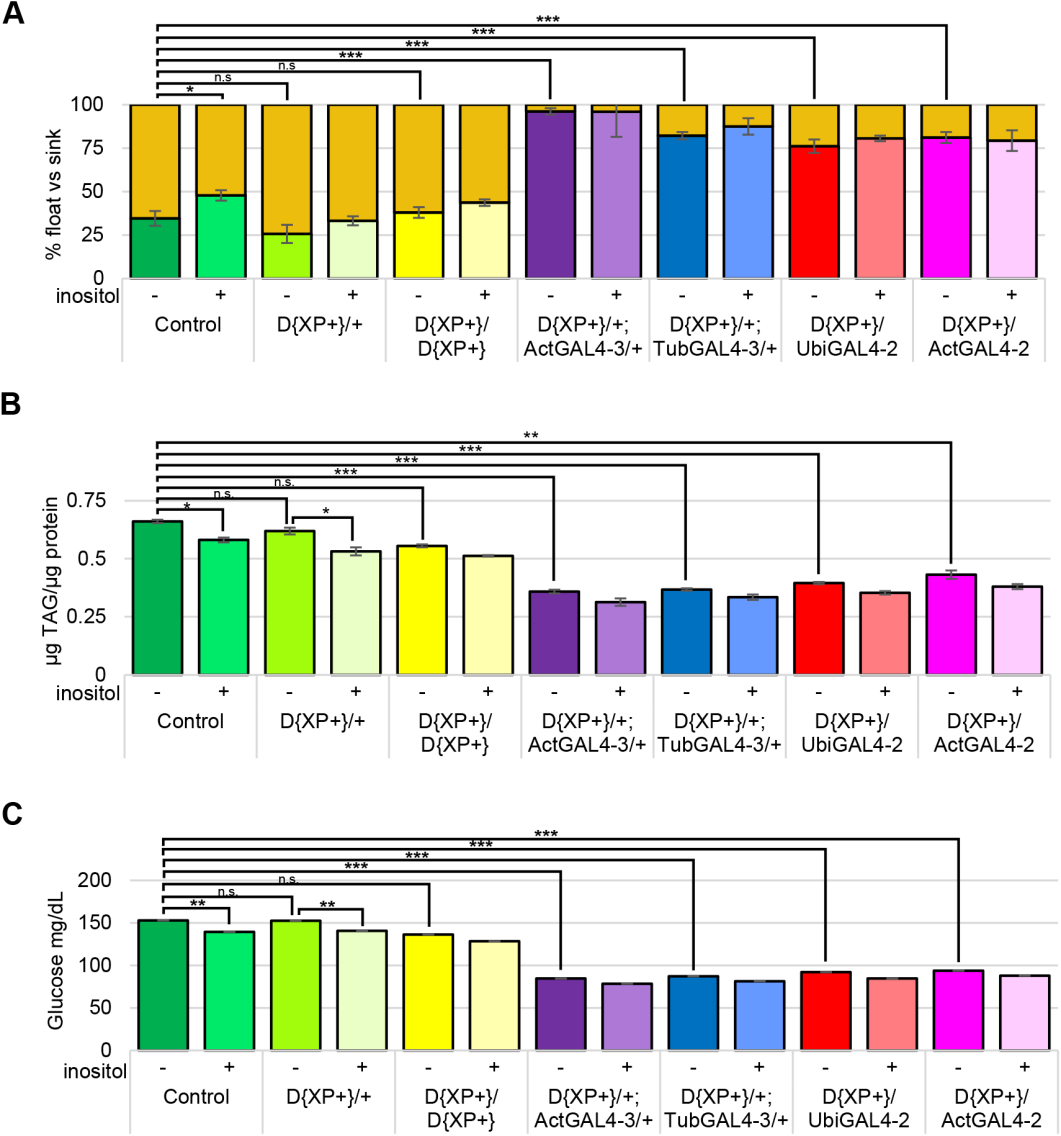
**Increased *Inos* mRNA levels decrease larval obesity and reduce hemolymph glucose.** Larvae grown on standard rich food with inositol supplementation as indicated. (A) The percentage of larvae of the indicated genotypes that float (gold segment of bars) and sink in a buoyancy assay are displayed. *N*=total number of larvae. Mean ±s.e. of four independent trials are represented. (B) Larvae assayed for TAG levels, values indicated are normalized to total protein. *N*=6 per condition per trial. Mean ±s.e. of three independent trials are represented. (C) Glucose (mg/dl) in the hemolymph of the larvae. *N*=5 per condition per trial. Mean±s.e. of three independent trials are represented. *P<0.05; **P<0.005; ***P<0.0001 as indicated determined by two-tailed *t*-test. Three independent trials of control strains ActGal4-3/+, TubGal4-3/+, UbiGal4-2/+, ActGal4-2/+ were indistinguishable from the wild-type control Canton-S results shown for all three experiments.

Consistent with the results of dietary inositol supplementation, the strains with dysregulated high levels of *Inos* mRNA and MIPSp had dramatically reduced proportions of obese-like (floating) larvae, decreased TAG levels, and lower hemolymph glucose levels (Fig. 4).

### Dysregulated increased *Inos* mRNA levels result in developmental defects

**Fig. 5.**
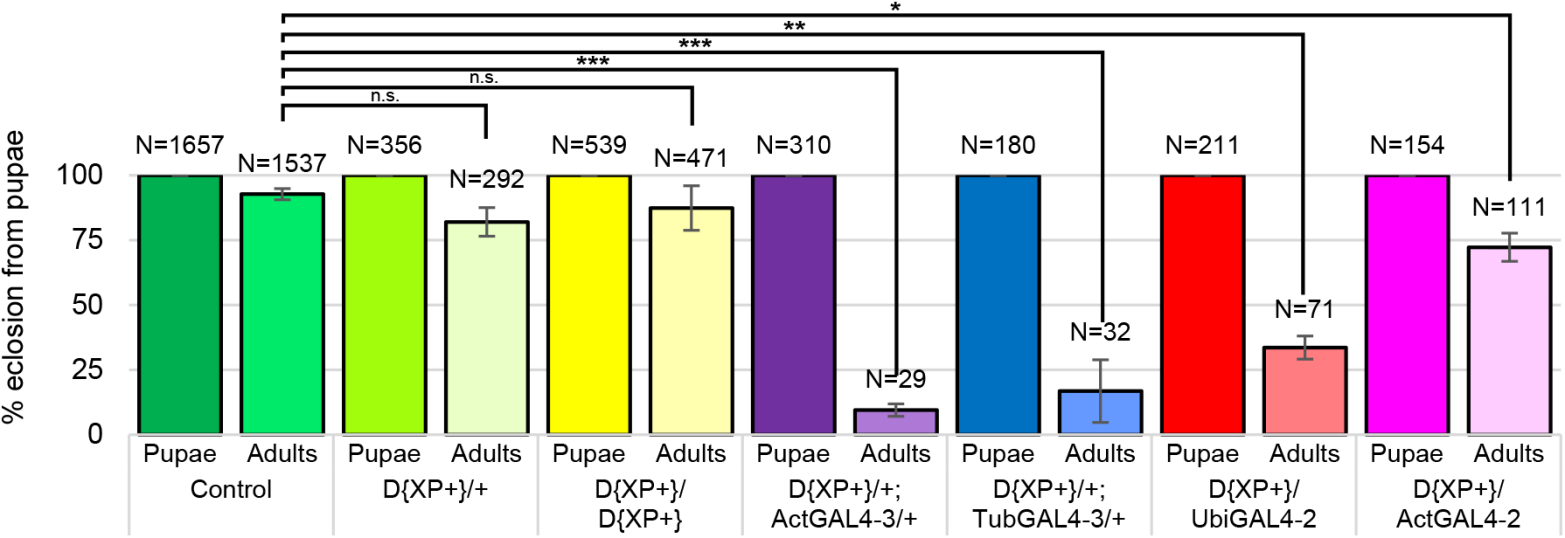
**Dysregulated increased *Inos* RNA levels result in developmental arrest as pharate adults.** The percent of adults (lighter bars) eclosing from pupae (darker bars) on standard rich food. Strains as indicated. Three independent trials of control strains ActGal4-3/+, TubGal4-3/+, UbiGal4-2/+ were identical to the wild-type control Canton-S results shown. *N*=total number of individuals examined. Mean±s.e. of three trials are represented. *P<0.05; **P<0.005; ***P<0.0001 as indicated determined by two-tailed *t*-test.

**Fig. 6.**
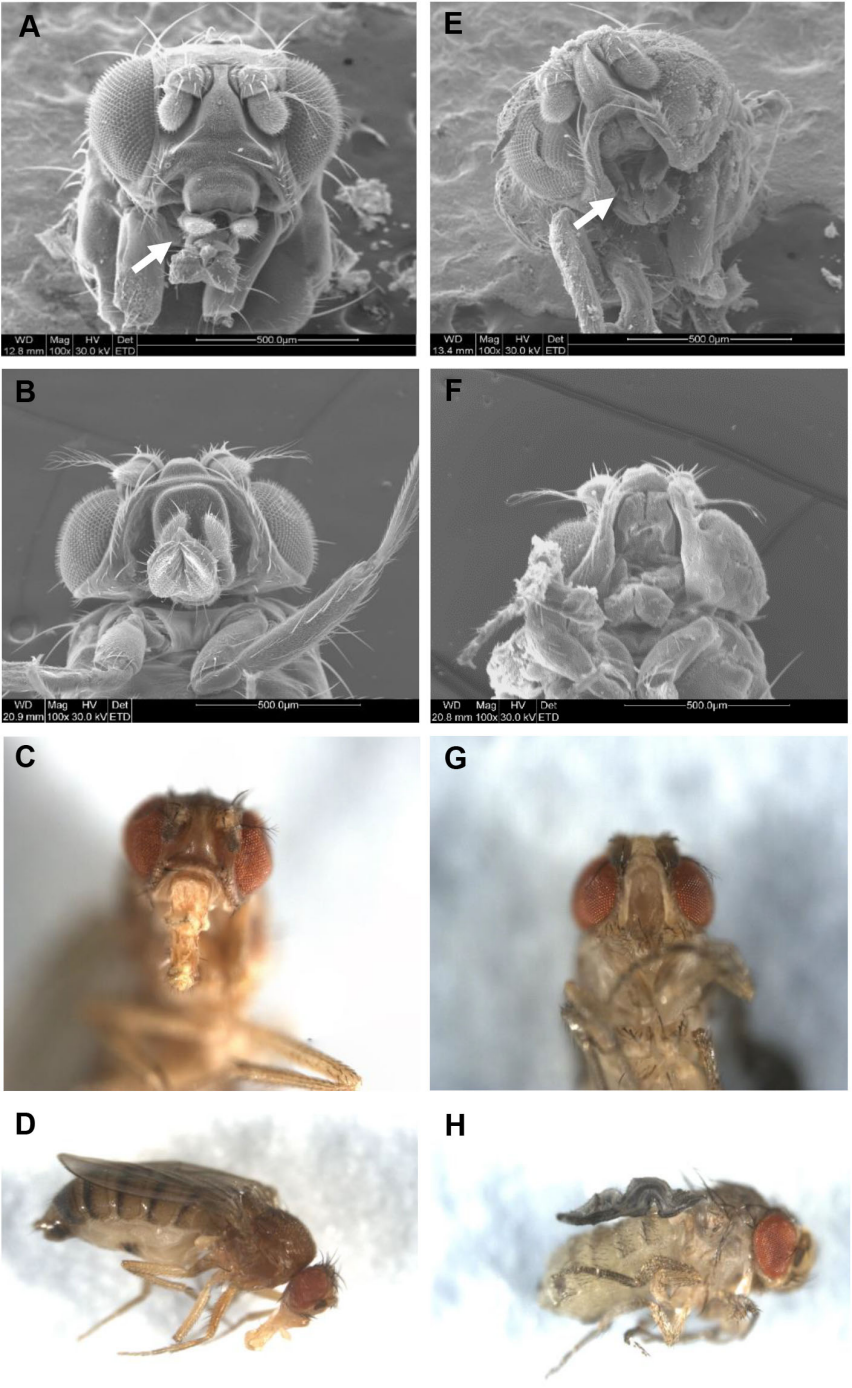
**Dysregulated increased *Inos* RNA levels result in developmental morphological defects.** Representative scanning electron (A,B,E,F) and brightfield (C,D,G,H) microscope images of adult females after eclosion. Panels A-D are wild-type control Canton-S (*N*=4 for A and B, *N*=10 for C and D) and panels E-H are D{XP+}/+; ActGAL4-3/+ (*N*=5 for E and F, *N*=15 for G and H). The arrows in A and E indicate the proboscis in the wild-type or the region lacking the proboscis in the genetically modified strain.

During these studies, it became apparent that dysregulation of *Inos* mRNA levels resulted in developmental abnormalities. Although differences in the rates of development and in viability are observed from embryogenesis through pupation, dramatic differences are apparent in the pupal to adult transition (Fig. 5). Fewer than 10% of the D{XP+}/+; ActGAL4-3/+ pupae eclosed as adults. Nearly all the flies died as pharate adults. Among hundreds examined, approximately two-thirds arrested growth at stage P8 and most of the remaining pupae arrested growth immediately before eclosion (stage P15). The few that eclosed showed a striking phenotype. As seen in Fig. 6 they lack a proboscis. Some structural alteration of wings and legs are also apparent. All the leg segments seem to be present, but the femora appear buckled. The wings appear twisted and fail to expand. In contrast, 71% of the D{XP+}/ActGAL4-2 pupae eclosed and all appeared phenotypically wild-type. The D{XP+}/ UbiGAL4-2 and D{XP+}/+_;_ TubGAL4-3/+ strains showed intermediate numbers of eclosing adults, but all showed proboscis defects similar to the D{XP+}/+; ActGAL4-3/+ (Fig. 6).

## DISCUSSION

Dietary inositol reduces *Inos* mRNA levels in wild-type Canton-S *Drosophila melanogaster* larvae grown on semi-defined low- or high-sucrose diets (Fig. 1). This seems to be the first report that *Inos* mRNA levels are regulated in response to dietary inositol in an animal. It was also observed that a high-sucrose diet reduced *Inos* mRNA levels, relative to a low-sucrose diet, independent of inositol.

This study also showed that dietary inositol alleviates obese-like and hyperglycemic phenotypes in wild-type Canton-S *D. melanogaster* third instar larvae raised on low- and high-sucrose diets as well as on standard rich food (Figs 2 and 4). As in prior studies, this study demonstrated that a high-sucrose diet can induce diabetic-like phenotypes in *D. melanogaster* larvae (Musselman et al., 2011). Dietary inositol was similarly shown to reduce fat deposits in mice fed a high-fat diet (Croze et al., 2015). These data parallel clinical trials demonstrating that dietary inositol reduces obesity in patients with gestational diabetes (D'Anna et al., 2019, 2021). Furthermore, type 2 diabetic patients had lowered fasting blood glucose and glycated hemoglobin when treated with dietary inositol (Pintaudi et al., 2016).

Similar to the results obtained with wild-type Canton-S larvae grown on semi-defined food, provision of 50µM dietary inositol reduces *Inos* mRNA levels in Canton-S larvae grown on standard rich food (Fig. 3B). To gain insight into the importance of *Inos* gene regulation, a strain harboring a ∼7 kb P-element 40 nucleotides upstream of the transcriptional start site (D{XP+}/D{XP+}) was examined (Fig. 3A). In contrast to wild-type Canton-S larvae, the level of *Inos* mRNA in homozygous (D{XP+}/D{XP+}) larvae was nearly constitutive (did not vary with dietary inositol) and was similar to that of wild-type larvae with 50 µM dietary inositol (Fig. 3B). It is interesting to note that there is no apparent alteration of development or growth of this homozygous strain.

The UAS_GAL4_ sequence in the D{XP+} element is in the correct orientation to promote transcription of the *Inos* gene when the GAL4p is present (Fig. 3A). Three different highly-expressed promoters (Actin 5C, Tubulin 84B, and Ubiquitin), on two different chromosomes, were used to express the *Saccharomyces cerevisiae GAL4* gene and control *Inos* transcription in heterozygous strains. In all the strains increased *Inos* mRNA levels were observed (Fig. 3B), which correspond to increased levels of the *Inos* gene product, MIPSp (Fig. 3C). Although higher than wild-type in all cases, a range of levels of *Inos* mRNA was observed in heterozygous third instar larvae with the D{XP+} element and the three different promoter-GAL4 constructs. In descending order from highest to lowest levels is the strain with the Actin 5C GAL4 construct on chromosome 3 (ActGAL4-3), the Tubulin 84B GAL4 construct on chromosome 3 (TubGAL4-3), the Ubiquitin GAL4 construct on chromosome 2 (UbiGAL4-2), and the Actin 5C GAL4 construct on chromosome 2 (ActGAL4-2). The differences seen in the levels of *Inos* mRNA with the ActGAL4 construct on chromosome 2 versus chromosome 3 appear to be due to position effects, as the promoter is the same (Small and Arnosti, 2020).

Data from float-buoyancy and TAG assays suggest that increased levels of *Inos* mRNA correspond to reduced obesity. The lowest proportion of obese-like (floating) larvae and lowest TAG levels were observed in strains with the highest levels of *Inos* mRNA (Figs 3 and 4). Specifically, these experiments revealed the exact same order (ActGAL4-3, TubGAL4-3, UbiGAL4-2, ActGAL4-2) of the heterozygous D{XP+} strains containing the GAL4 constructs. Moreover, an examination of the levels of hemolymph glucose revealed that increased levels of *Inos* mRNA correspond to decreased hemolymph glucose. Once again, the exact same order (ActGAL4-3, TubGAL4-3, UbiGAL4-2, ActGAL4-2) of the heterozygous D{XP+} strains containing the GAL4 constructs was observed, with lower levels of hemolymph glucose in strains with higher levels of *Inos* mRNA (Figs 3 and 4).

Experiments examining the development and growth of these strains revealed dramatic differences in the proportion of the pupae eclosing as viable adult flies. The exact same order (ActGAL4-3, TubGAL4-3, UbiGAL4-2, ActGAL4-2) of the heterozygous D{XP+} strains containing the GAL4 constructs was again observed, with the D{XP+}/+; ActGAL4-3/+ strain producing the fewest viable adults (Fig. 5). These data suggest that increased levels of *Inos* mRNA correspond to reduced viability during the pupal to adult transition. Mutations in many genes that affect imaginal disc development are known to cause pupal lethality. One example is flies with mutations in the trithorax group gene *little imaginal discs* (*lid*), many of which died as early pupae with the rest dying as pharate or newly eclosed flies (Gildea et al., 2000). However, comparison of the eye antennal and wing discs isolated from third instar larvae of wild-type Canton-S and the heterozygous D{XP+} strains containing the GAL4 constructs revealed no gross morphological defects (data not shown). Therefore the arrested pupae were examined in more detail. These further analyses demonstrated that there were two stages of developmental arrest as pharate adults, early (pupal stage 8) and late (pupal stage 15) (Bainbridge and Bownes, 1981).

Pupal lethality has also been reported in *D* *melanogaster* strains harboring mutations in numerous other pathways, with a consensus that the arrests often appear to be caused by events that occurred earlier in development (Shearn et al., 1971; Engels et al., 1987; Gildea et al., 2000; Yoshida et al., 2016; Kowalewski-Nimmerfall et al., 2014; Rosales-Vega et al., 2018; Syed et al., 2019; Drelon et al., 2019; Ray and Lakhotia, 2019; Wang et al., 2019; Yusuff et al., 2020). Having established that inositol is causing developmental defects, some next steps would be to determine if this is due inositol's role in membrane biogenesis, signal transduction, osmoregulation, as a carbon/energy source, as an insulin mimetic, or in some other component of maintenance of homeostasis and/or metabolism. Since inositol is a precursor for the phosphatidylinositol phosphates (PIPs), it is interesting to note that dysregulation of the expression of the *phosphatidylinositol synthase* gene (*Pis*) causes pupal lethality (Janardan et al., 2020). *Inositol phosphate kinase 2* (*Ipk2*) deletion mutants also show pupal lethality (Seeds et al., 2015). Moreover, temperature sensitive mutations in the *Sac1* phosphoinositide phosphatase gene have been shown to cause ectopic *dpp* expression and display embryonic and pupal lethality, and downregulation of *Sac1* specifically in the nervous system also results in failure to eclose (Wei et al., 2003; Forrest et al., 2013; Del Bel and Brill, 2018; Janardan et al., 2020). Although the developmental defects observed in this study could be caused by many different mechanisms, it is tantalizing to speculate that the inositol metabolic and the insulin pathways converge. Drosophila insulin-like peptide 8 (dILP8) has been shown to provide the signal for the larval to pupal transition whereby decreased levels of dILP8 lead to synthesis of the steroid hormone ecdysone (Colombani et al., 2012; Garelli et al., 2012; Delanoue and Romero, 2020). Ray and Lakhotia (2019) have shown that mutant fly strains with high levels of dILP8 in early pupae can inhibit the post-pupation ecdysone surge resulting in pupal lethality.

Pupal lethality is not an unusual trait. In this study, however, the few experimental flies that eclosed lacked proboscises and had defective wings and legs (Fig. 6). All the experimental flies that eclosed died within 2 days, probably due to the severity of the morphological defects. Often eclosion of the experimental flies, but not the control flies, was delayed by several days (data not shown).

The cumulative amount of inositol is determined by synthesis, transport, recycling, and catabolism. This study demonstrates that organisms usually regulate inositol synthesis levels within a range that can be exceeded to a certain extent causing reduced viability but no apparent morphological defects. Beyond this level, increases in inositol synthesis cause dramatic morphological defects as well as reduced viability. The use of different promoters driving GAL4 production enabled an examination of the effects of different levels of *Inos* mRNA. With the Actin 5C promoter on chromosome 2 (ActGAL4-2), reduced viability is evident but there are no apparent morphological defects. With the ubiquitin promoter the level of *Inos* mRNA is only slightly higher than with ActGAL4-2 and there is a further loss of viability (Figs 3 and 5). Surprisingly, in contrast to organisms with ActGAL4-2 promoter, the rare pharate or newly eclosed flies with the ubiquitin promoter display dramatic morphological defects. Further increases in *Inos* mRNA levels, with GAL4 production driven by the Tubulin 84B and Actin 5C promoters on chromosome 3 resulted in even further losses in viability but no additional morphological defects. It is interesting to note that the same Actin 5C promoter resulted in vastly different effects on viability and morphology, coincident with the difference in levels of *Inos* mRNA expression.

Overall it appears that the level of *Inos* mRNA, and not the metabolic, temporal, or spatial dysregulation, causes the observed developmental defects. Although reminiscent of phenotypes observed with mutations in the head involution defective *(hid*) (Abbott and Lengyel, 1991) and decapentaplegic (*dpp*) genes (Hursh and Stultz, 2018; Setiawan et al., 2018; Simon et al., 2019), the phenotypes caused by the overexpression of the *Inos* gene are unique and do not appear to have been previously described in the literature. This study, at the junction of metabolism and development, furthers the understanding of the importance of regulated inositol synthesis and may have implications in the treatment of diabetes and neurodegenerative disorders.

## MATERIALS AND METHODS

### Fly stocks and maintenance

Flies were maintained in standard laboratory conditions at 18°C or 25°C and 70-80% humidity on a 12 h:12 h light-dark cycle. Stocks from the Bloomington Drosophila Stock Center include Canton-S (#1, CS), *w^−^; sna*^Sco^ / CyO, S*bw^1^ P{*Act*GFP *w^−^* }CC2 (#9325, hereafter identified as CyO^GFP^), w[1118]; Df(3L)Ly,sens[Ly-1]/TM6B, P{w[+mW.hs]=UbiGFP.S65T}P82,Tb[1] (#4887, hereafter identified as Tb^GFP^), w[1118]; P{w[+mC]=AyGAL4}25/CyO (#3953, hereafter identified as ActGAL4-2/CyO), y[1] w[*]; P{w[+mC]=Act5C-GAL4}17bFO1/TM6B, Tb[1] (#3954, hereafter identified as ActGAL4-3/Tb), y[1] w[1118]; P{w[+mC]=UAS-mCD8::GFP.L}LL6, P{w[+mC]=tubP-GAL4} LL7/TM3, Sb[1] (#30030, hereafter identified as TubGAL4-3/Sb), and w[*]; P{w[+m*]=Ubi-GAL4.U}2/CyO (#32551, hereafter identified as UbiGAL4-2/CyO). Stock from Exelixis Harvard Medical School is w^−^; P{XP}d00881 (#D00881, hereafter identified as D{XP+}). Heterozygotes are identified as D{XP+}/+ or other markers.

All fly stocks were grown on either rich food (BDSC cornmeal food, https://bdsc.indiana.edu/information/recipes/bloomfood.html) or modified semi-defined food (per liter 10 ***g*** agar (ThermoFisher Scientific), 80 ***g*** brewers yeast (Genesee), 20 ***g*** yeast extract (ThermoFisher Scientific), 20 ***g*** peptone (ThermoFisher Scientific), sucrose (ThermoFisher Scientific) as indicated, Musselman et al., 2011) with or without 50 µM inositol (Sigma-Aldrich) as indicated (this concentration is sufficient to support growth of a homozygous *Inos* deletion mutant (inos^ΔDF^/inos^ΔDF^), Jackson et al., 2018).

### RNA extraction and qRT-PCR

Total RNA was extracted from 10–20 third instar larvae or adult flies grown on the food indicated using Trizol™ (Life Technologies) (Green and Sambrook, 2012). Total RNA (1 µg) was DNase treated using the DNA-free Kit (Ambion) and inactivation buffer (DNA-free DNA Removal Kit, Invitrogen). cDNAs were generated using oligo (dT) 18 primers (Eurofins), dNTPs (ThermoFisher Scientific), and Moloney Murine Leukemia Virus Reverse Transcriptase (M-MLV RT) (Fisher). After amplification the samples were treated with RNAse H (New England BioLabs). The cDNA was diluted in RNase/DNase free water (ThermoFisher Scientific) (1:16) and for qRT-PCR experiments using ThermoFisher Scientific Absolute qPCR Mix, SYBER Green, ROX (Fisher) in an Applied Biosystems StepOnePlus System. Triplicate samples were used in all the experiments including linearizations and melt curves. All the experiments were performed at least three independent times (separate biological samples) as indicated in the figure legends. The results were normalized to the transcript levels of ribosomal protein 32 (RpL32). The following primers were used: *Inos* exon 1-2 forward GAAAGTGCAGGTGGACGATG and reverse GTCAGCGTGGATCCGTTGT, RpL32 forward CCAGCATACAGGCCCAAGAT and reverse GCACTCTGTTGTCGATACCCT.

### Float buoyancy assay

The float buoyancy experiments were conducted essentially as described by Reis et al. 2010 and Reis 2016 using 30-50 larvae per sample, with initial results confirmed by the method of Hazegh and Reis (2016). A relationship between the percentage of larvae floating in a buoyancy assay and the percent body fat of the larvae has been established (Reis et al., 2010; Tsuda-Sakurai et al., 2015). Thus, in this study obesity is defined by the relative proportion of the larvae floating in the buoyancy assay.

### TAG Assay

Total TAG concentration was measured using the Serum Triglyceride Determination Kit (TR0100, Sigma-Aldrich) and Triglyceride Reagent (T2449, Sigma-Aldrich) essentially as described by Tennessen et al. (2014). Six third-instar larvae per replicate were homogenized in PBS with 0.1% Tween.

### Hemolymph glucose assay

These experiments were performed essentially as described by Tennessen et al. (2014). Hemolymph was collected by puncturing five third instar larvae and the Sigma-Aldrich Glucose (GO) Assay Kit GAGO-20 was used.

### Protein extraction and western blots

Protein concentration of crude lysates, homogenates of 20-30 third instar larvae, were determined using the Bradford Assay (Bradford, 1976) with bovine serum albumin (Pierce™ Bovine Serum Albumin Standard, ThermoFisher Scientific) and using a dye concentrate (5000006, Bio-Rad) for the colorimetric analysis. Thirty µg of crude lysates, and pre-stained protein size standard (EZ Run Prestained Rec Protein Marker, Broad Range 10-175 kDa ThermoFisher Scientific), were loaded on 10% SDS-acrylamide gels. The proteins were transferred to nitrocellulose filters (BioRad Trans-Blot Turbo 1704156) using the Abcam protocol (http://www.abcam.com/protocols/general-western-blot-protocol) and the BioRad Trans-Blot Turbo Transfer system. The blots were stained with Ponceau S (ThermoFisher Scientific) according the protocol of Romero-Calvo et al. (2010). Western blots were performed using a polyclonal rabbit anti-human *myo*-inositol-3-phosphate synthase antibody (ThermoFisher Invitrogen Catalog # PA5-44105; diluted 1:1000) and anti-rabbit IgG alkaline phosphatase conjugated secondary antibody (Promega; diluted 1:7500). Alkaline phosphatase activity was detected using Nitro Blue Tetrazolium Chloride (0.34 mg/ml), and 5-Bromo-4-Chloro-3-Indolylphosphate p-Toluidine Salt (0.17 mg/ml).

### Pupariation and Eclosion

Female and male adults (2:1) were placed in vials of standard rich food in a 25°C incubator at 70-80% humidity on a 12 h:12 h light:dark cycle. The progeny (embryos) were sorted using the GFP marker, and reconfirmed as larvae. The vials were checked daily for up to 28 days, to allow sufficient time for genotypes with developmental delays to eclose. Staging of the pupae were based on images and descriptions in Bainbridge and Bownes (1981). The number of pupae were recorded, as was the number of adults that eclosed.

### Scanning electron and light microscopy

Samples were fixed in glutaraldehyde, dehydrated in an ascending series of ethanol, critical point dried using a Samdri-PVT-3D (Tousimis, Rockville, MD, USA), mounted on a metallic stub using conductive carbon tape, and sputter coated with gold and palladium. Scanning electron micrographs (SEM) of four control and five experimental independent specimens were taken with a SEM/FEI Quanta 200 supported by the software. Ten control and fifteen independent experimental specimens were fixed and viewed on a Nikon SMZ1500 microscope and images were captured using a Micropublisher six-color CCD camera system (Teledyne Q imaging). Sample sizes are indicated in the results section.

### Statistical analyses

Standard error was calculated for all experiments. The *p*-values of pairwise comparisons were determined using student's two-tailed *t*-test.
